# Methodologies to unlock the molecular expression and cellular structure of ocular lens epithelial cells

**DOI:** 10.3389/fcell.2022.983178

**Published:** 2022-09-13

**Authors:** Justin Parreno, Grace Emin, Michael P. Vu, Jackson T. Clark, Sandeep Aryal, Shaili D. Patel, Catherine Cheng

**Affiliations:** ^1^ Department of Biological Sciences, University of Delaware, Newark, DE, United States; ^2^ Department of Biomedical Engineering, University of Delaware, Newark, DE, United States; ^3^ School of Optometry and Vision Science Program, Indiana University, Bloomington, IN, United States; ^4^ Department of Neurology, Massachusetts General Hospital and Harvard Medical School, Boston, MA, United States

**Keywords:** Western blot, flat mount, RNA, PCR, primary culture, immunostaining, whole mount

## Abstract

The transparent ocular lens in the anterior chamber of the eye is responsible for fine focusing of light onto the retina. The lens is entirely cellular with bulk of the tissue composed of fiber cells, and the anterior hemisphere of the lens is covered by a monolayer of epithelial cells. Lens epithelial cells are important for maintaining fiber cell homeostasis and for continual growth of the lens tissue throughout life. Cataracts, defined as any opacity in the lens, remain the leading cause of blindness in the world. Following cataract surgery, lens epithelial cells can undergo a process of epithelial-to-mesenchymal transition (EMT), leading to secondary cataracts due to posterior capsular opacification (PCO). Since the epithelial cells make up only a small fraction of the lens, specialized techniques are required to study lens epithelial cell biology and pathology. Studies using native lens epithelial cells often require pooling of samples to obtain enough cells to make sufficient samples for traditional molecular biology techniques. Here, we provide detailed protocols that enable the study of native mouse lens epithelial cells, including immunostaining of the native lens epithelium in flat mounts, extraction of RNA and proteins from pairs of lens epithelial monolayers, and isolation of lens epithelial cells for primary culture. These protocols will enable researchers to gain better insight on representative molecular expression and cellular structure of lens epithelial cells. We also provide comparative data between native, primary culture, and immortalized lens epithelial cells and discuss the advantages and disadvantages of each technique presented.

## Introduction

The eye lens is an ellipsoid, transparent tissue in the anterior chamber of the eye that is responsible for fine focusing light onto the retina to create a clear image (Lovicu and Robinson, 2004). The bulk of the lens is composed of fiber cells, while a monolayer of the lens epithelium covers the anterior hemisphere of the lens (Piatigorsky, 1981). The entire tissue is encapsulated by a basement membrane, known as the lens capsule (Lovicu and Robinson, 2004). Lens epithelial cells are divided spatially into anterior epithelial cells that are quiescent and equatorial epithelial cells that continuously proliferate, migrate, elongate, and differentiate into new generations of lens fiber cells throughout life (Piatigorsky, 1981; Kuszak et al., 2004; Yamamoto et al., 2008). Anterior epithelial cells are cuboidal and cobblestone in cross-section, while equatorial epithelial cells transition to the organized hexagon-shaped cells at the region of epithelial-to-fiber cell differentiation (Bassnett et al., 1999; Zampighi et al., 2000; Cheng and Gong, 2011; Cheng et al., 2013).

Lens epithelial cells are oriented with their basal side toward the capsule. These cells have a strong attachment to the capsule, while the apical–apical junction with fiber cells is a relatively weaker adhesion (Wang et al., 2007). The interface between epithelial and fiber cells creates a rare apical–apical junction within the tissue (Zampighi et al., 2000). Fiber cells are continuously added to the lens in concentric shells with the newly formed cells on the periphery (Piatigorsky, 1981), similar to rings on a tree. As fiber cells mature and compact, they lose all of their cellular organelles to reduce light scattering (Bassnett et al., 2011). These cells become highly compact at the center of the lens, known as the lens nucleus. Anterior epithelial cells are thought to be important for cellular communication and maintenance of underlying mature fiber cells (Lovicu and Robinson, 2004). In rodent lenses, the nucleus is compact and can be separated from cortical fibers by dissection (Gong et al., 1997; De Maria et al., 2009), controlled lysis (Pierscionek and Augusteyn, 1988; De Maria et al., 2009; Cheng et al., 2022), or mechanical separation (Gokhin et al., 2012; Cheng et al., 2016; Cheng et al., 2018; Cheng et al., 2019; Cheng et al., 2022). Since cell proliferation is restricted to lens equatorial epithelial cells, continual differentiation of lens epithelial cells to fiber cells for lifelong lens growth is dependent on lens epithelial cells (Piatigorsky, 1981; Shi et al., 2014).

Specialized techniques are required to study the limited population of lens epithelium monolayer cells. Lens epithelial cell differentiation and behavior have been studied in many species using *in vitro* primary cell culture and immortalized cell lines (Kirby, 1927; Mann, 1948; Mamo and Leinfelder, 1958; Van Der Veen and Heyen, 1959; Okada et al., 1971; Creighton et al., 1976; Hamada and Okada, 1978; Reddan et al., 1980; Menko et al., 1984; FitzGerald and Goodenough, 1986; Reddan et al., 1989; Musil et al., 1990; Bermbach et al., 1991; Andley et al., 1998; Ibaraki et al., 1998; Ogiso et al., 1998; Wang et al., 2007; Bannik et al., 2013; Matsuyama et al., 2013; Sundelin et al., 2014; Terrell et al., 2015; Wang et al., 2017; Weatherbee et al., 2019). However, primary lens epithelial cells in culture often do not maintain native lens epithelial cell characteristics. Examining lens epithelial cell behavior in native lenses is challenging. Protein and nucleic acid isolation of whole lenses disproportionately reflect molecular expression from fiber cells, which make up the bulk of the lens. Separation of epithelial cells from fiber cells to enrich epithelial cell isolates is possible; however, traditional methods of RNA extraction and protein analysis (Western blotting) require the pooling of multiple lens epithelia to increase extraction yield. Additionally, the examination of lens tissue sections often focuses on fiber cells and does not reveal changes in the epithelial monolayer that is reduced to a 2D cross-section of the monolayer. Therefore, refined methods are required for in-depth study of lens epithelial cell changes due to aging, genetic manipulation, or diseases.

We describe in detail several methods to study the lens epithelial cells from mouse tissue, including 1) primary culture, 2) RNA isolation, 3) protein extraction, and 4) immunostaining of flat mounts of lens epithelial cell sheets and whole-mount lenses.

1) Primary culture isolation of epithelial cells has been performed by many other researchers from various animals, including mice (Mann, 1948; Reddan et al., 1989; Andley et al., 1998; Wang et al., 2007; Bannik et al., 2013; Wang et al., 2017). The detailed primary epithelial cell isolation method presented here is our modification of a previously published method (Wang et al., 2007).
2) The method for RNA isolation presented here is a novel protocol for lens epithelial cells. We present method validation data by comparing RNA expression levels for various epithelial and mesenchymal markers between native epithelial cells, primary culture cells, and immortalized cells.
3) We detail our previously published method for protein isolation from lens epithelial cells (Parreno et al., 2020; Cheng et al., 2022), cortical fiber cells, and nuclear fiber cells (Cheng et al., 2022), including homogenization buffer volumes and the expected protein yield for each fraction. We compare β-actin levels between the three protein fractions isolated from the lens.
4) To better visualize the lens epithelial cell monolayer, we also present a detailed protocol to immunostain lens capsule flat mounts with attached epithelial cells (Cheng and Gong, 2011; Cheng et al., 2013; Cheng et al., 2017). We include crucial details to distinguish the anterior and posterior poles of the lens and show continuous imaging of the epithelial monolayer from the anterior pole to the equator to compare cell arrangement and shape from distinct regions of the lens.

Our methods for protein and RNA extraction can be applied to single or pairs of lens epithelial monolayers, reducing the number of animals required for generating these samples and allowing researchers to produce more biological replicates for improved statistical analysis. In general, the procedures described here use common laboratory equipment. However, protein analysis of epithelial cell lysates requires a specialized instrument for capillary-based electrophoresis (Parreno et al., 2020; Cheng et al., 2022). Using capillary-based electrophoresis allows examination of protein expression from single or paired mouse lens epithelium. While the methods we present here were developed for mouse lenses, they could be adapted for lenses from larger animals or humans.

### Step-By-Step Procedures

All procedures were conducted in accordance with the approved animal protocols from the University of Delaware and Indiana University, Bloomington Institutional Animal Care and Use Committees (IACUC) and in accordance with the Use of Animals in Ophthalmic and Vision Research and the Guide for the Care and Use of Laboratory Animals by the National Institutes of Health. A video of, and a detailed protocol for, mouse lens dissection can be found in our previous work (Cheng et al., 2016). Dissection of freshly enucleated eyes was performed in 1X phosphate-buffered saline (PBS) in a dissection plate. PBS for dissection is maintained at room temperature, but for lenses from mice younger than postnatal day 14 (P14), PBS should be warmed to 37°C to avoid cold cataracts (Zigman and Lerman, 1964; Lo, 1989; Wang et al., 2007). A complete material list is provided in Supplementary Table S1.

#### Lens epithelium and fiber mass separation for epithelial cell isolation for primary culture, RNA isolation, and protein extraction

1) For protein and RNA isolation, any tissue stuck to the outside of the dissected lens is removed by transferring the lens to a Kimwipe using curved forceps and gently rolling the lens along the Kimwipe. This will remove most/all small tissue bits that may be adhered to the lens capsule. This step may be repeated until the lens capsule is completely free of visible extraneous tissues.

It should be noted that after rolling on the Kimwipe, the capsule may become slightly hazy due to brief drying. The capsule should become clear again after the lens is rehydrated in the next step. For RNA isolation, spraying RNAse away on the Kimwipe will help reduce the RNA degradation caused by RNAse contamination.

2) The clean lens is transferred back to the dissection dish filled with 1X PBS. The lenses are weighed to obtain the whole lens wet weight if needed to determine the amount of buffer to be used in subsequent steps.
3) Using the fine straight forceps, the capsule is carefully punctured at the lens equator and the capsule is peeled off in one continuous sheet. The lens epithelial cells are firmly attached to the lens capsule. The mass of the lens fiber cells will remain intact. The capsule with the attached lens epithelial cells is transferred to the appropriate buffer for the next step of the specific experiment.

#### Isolation of lens epithelial cells for primary culture

**FIGURE 1:**
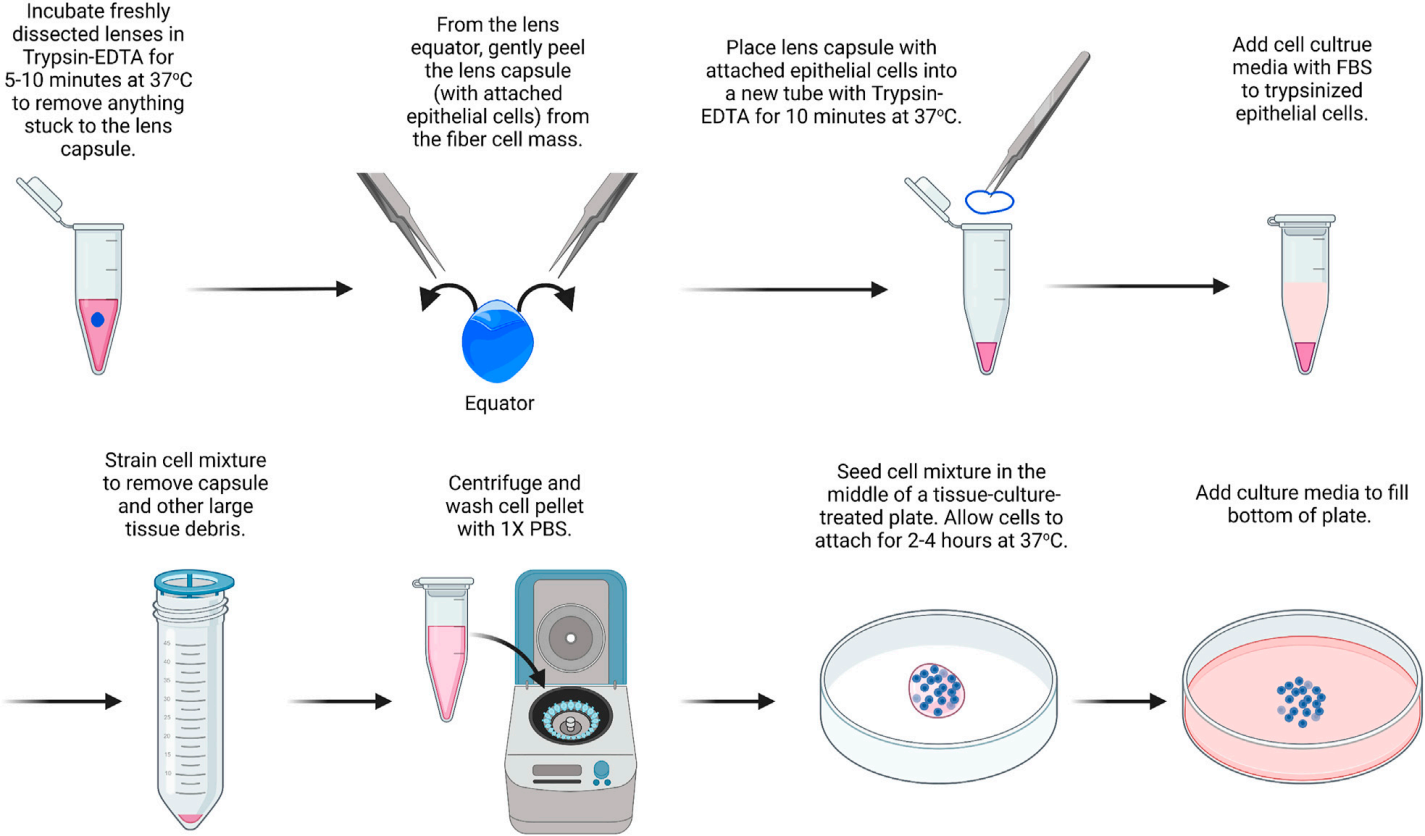
Primary lens epithelial cell isolation. Lenses are placed in trypsin/EDTA solution to remove extraneous materials that could stuck to the lenses during dissection. Capsules are then isolated and placed in trypsin/EDTA to liberate/dissociate cells. Cells are seeded onto the center of a culture dish and filled with media after they attach. The image was drawn using Biorender and not drawn to scale.

For this protocol (Figure 1), we suggest using lenses from P10 mice, which contain a substantial number of epithelial cells. This method is modified from a previous study (Wang et al., 2007). For this, we pool together 14 capsules. All solutions should be prepared under sterile conditions, and instruments should be autoclaved or brand new. Cultured cells should be cared for with regular medium changes. The cells will reach confluence in the center of the plate within about 14 days of culture and cannot be passaged. Instead of using a 35-mm dish, culture can also be carried out on chamber slides or in multi-well plates.

1) Place the dissected lenses in a 15-ml tube with 5 ml of 0.05% Trypsin-EDTA.

It should be noted that there is no need to remove small pieces of tissue attached to the capsule of the lens since the next step will remove any extraneous tissues. The blood vessels attached to the lenses from P14 or younger mice are difficult to remove by dissection without damaging the lens.

2) The lens is incubated in trypsin for 5–10 min at 37°C. Gently swirl the lenses in the trypsin solution every 1–2 min. Meanwhile, 0.4 ml of trypsin is prepared in another 1.5-ml microcentrifuge tube in sterile conditions for step 5.
3) 5 ml of Dulbecco’s modified Eagle’s medium (DMEM) containing 10% fetal bovine serum (FBS) is added to inactivate the trypsin.
4) The lenses are transferred to a clean plastic dish, and the lens capsule is dissected by puncturing the lens at the equator and using fine forceps to gently peel away the lens capsule with the attached lens epithelial cells.
5) The dissected lens capsules are placed in the 1.5-ml microcentrifuge tube with 0.4 ml of 0.05% Trypsin-EDTA.
6) The lens capsules are incubated in Trypsin-EDTA at 37°C for 10 min.
7) In a tissue culture hood, under sterile conditions, a cell strainer (100 µm) is added on top of a 50-ml tube. The lens capsule and trypsin mixture are passed through the cell strainer, collecting the cell sample at the bottom of the 50-ml tube. The cell strainer will help remove debris from the lens capsule so that only epithelial cells are left behind in the sample after filtering.
8) The cell sample is centrifuged at 800 × g for 8 min.
9) The supernatant is removed without disturbing the cell pellet. The cell pellet is resuspended in 1 ml of PBS and centrifuged again. Repeat PBS wash and centrifugation two more times.
10) The cell pellet is resuspended in 50 μL of media and seeded in the middle of a 35-mm tissue-culture treated plastic or glass-bottom dish.
11) Let the cells sit undisturbed at 37°C to allow for cell attachment. After approximately 2–4 h, the dish is filled with 2 ml of DMEM containing 10% FBS and 1% antibiotic/antimycotic.

The media is changed every 2–3 days, and the cells at the center of the plate will reach confluence in ∼7–10 days of culture.

#### RNA isolation from epithelial cells

**FIGURE 2:**
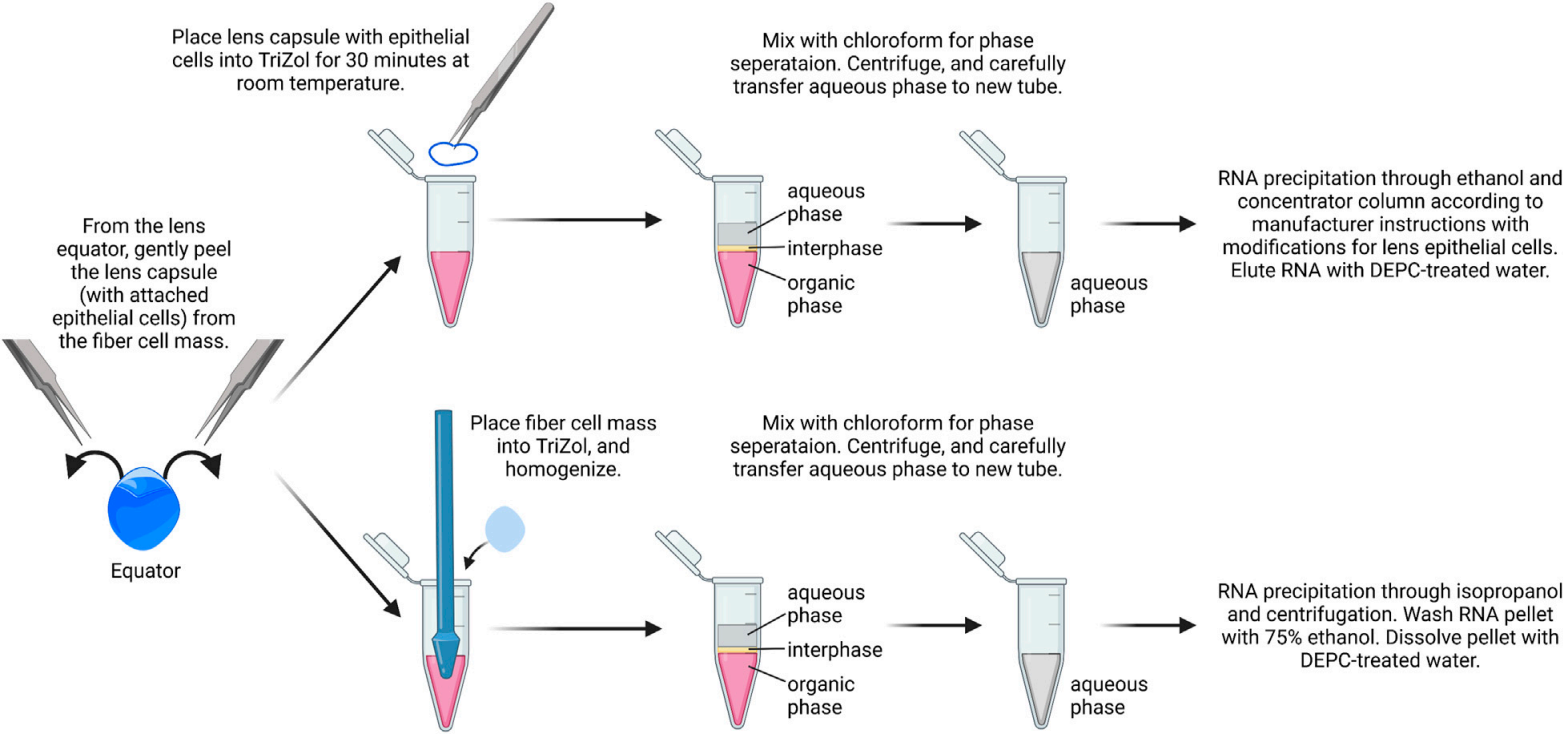
RNA isolation from lens epithelial cells. Capsules are isolated from lenses and placed in TRIzol. Chloroform is added for phase separation, and RNA is precipitated using ethanol followed by RNA clean up using spin columns. From the same samples, the fiber cell RNA can also be isolated with standard TRIzol/chloroform extraction, isopropanol precipitation, and ethanol wash. The image was drawn using Biorender and not drawn to scale.

While it is possible to isolate RNA from a single-lens capsule with attached epithelial cells, the yield is low in our experience. We recommend using a pair of capsules from the same mouse as one biological replicate. Typically, the RNA yield from a pair of lens capsules is ∼75–150 ng/μL (Figure 2). RNA from the fiber cell mass can also be isolated from the dissected lenses using a standard TRIzol–chloroform method. While we do not include that protocol here, we recommend using 20 µL of DEPC-treated water to dissolve the RNA pellets from pairs of fiber masses. Usually, the RNA yield from a pair of fiber masses is 500–800 ng/μL.

1) The lens capsule is placed immediately in 0.4 ml of cold TRIzol reagent to a 1.5-ml microcentrifuge tube. Tubes are inverted and ensure capsules are fully immersed in TRIzol.
2) Samples are incubated for 30 min at room temperature in a chemical fume hood for the lens capsule to fully dissolve.
3) For phase separation, 0.2 ml of chloroform is added per 0.4 ml of TRIzol added to act as a chemical cabinet. The tubes are shaken vigorously by hand for 15 s or vortexed lightly. Do not over vortex to avoid breaking up of RNA. Samples are incubated at room temperature for 10–15 min.
4) The sample is centrifuged at 14,000 x *g* for 15 min at 4°C. Transfer aqueous phase to a fresh tube using a P200 pipettor. Typically, there will be ∼150 µL of the aqueous phase. Avoid capturing the white protein layer between the aqueous phase and the pink TRIzol layer. Some of the aqueous layer will be left behind to avoid disturbing the white protein layer.

TRIzol and chloroform waste are collected in the phenol and the chloroform waste bottle. Leave them open in the fume hood overnight to allow for evaporation.

5) In a fresh tube with the aqueous layer only, an equal volume of 100% ethanol is added to the aqueous phase. The tube is gently inverted to gently mix the contents.

For steps 6–16, this protocol uses a ZYMO Research RNA extraction and a clean kit.

6) Transfer the entire volume of the sample to a new spin column with a collecting tube from the kit (e.g., if 150 μL of the top aqueous phase is collected, there will be a total of 300 μL of sample with an equal volume of ethanol added).
7) Centrifuge the column and collecting tube at 16,000 × g for 30 s at 4°C and remove the collected liquid from the collecting tube and dispose.

The RNA binds to the silica-based matrix at the bottom of the spin column tube, so pouring out the collected liquid does not remove the RNA.

8) Wash the RNA by adding 400 μL of RNA prep buffer into the spin column to remove impurities.
9) Centrifuge the column and collecting tube at 16,000 × g for 30 s at 4°C and discard the liquid from the collecting tube and dispose.
10) Add 700 μL of RNA wash buffer to the spin column tube.
11) Centrifuge the column and collecting tube at 16,000 × g for 30 s at 4°C and remove the collected liquid from the collecting tube and dispose.
12) Add 400 μL of RNA wash buffer to the spin column tube.
13) Centrifuge the column and collecting tube at 16,000 × g for 1 min at 4°C and discard the liquid from the collecting tube and dispose.
14) Centrifuge the column and collecting tube at 16,000 × g for 30 s at 4°C to remove any residual wash buffer and dispose the liquid from the collecting tube.
15) Replace the collecting tube with an RNAse-free 1.5-ml microcentrifuge tube and place the spin column on top of the new microcentrifuge tube.
16) Add 15 µL of DEPC-treated or molecular grade water directly onto the membrane of the spin column. Let water stand on the spin column membrane for 2 min, and then centrifuge the spin column and 1.5-ml microcentrifuge tube at 16,000 × g for 1 min at 4°C.
17) The collected ∼15 µL of sample is in the 1.5-ml microcentrifuge tube. This is the RNA sample.
18) Before quantifying, the RNA sample tube is incubated at 55–65°C for 10 min to dissolve the RNA. After this, the tubes are immediately placed on ice.
19) The RNA samples are stored at −80°C for future work as RNA is less stable at higher temperatures and will degrade.

#### Protein extraction from epithelial cells, cortical fibers, and nuclear fibers

**FIGURE 3:**
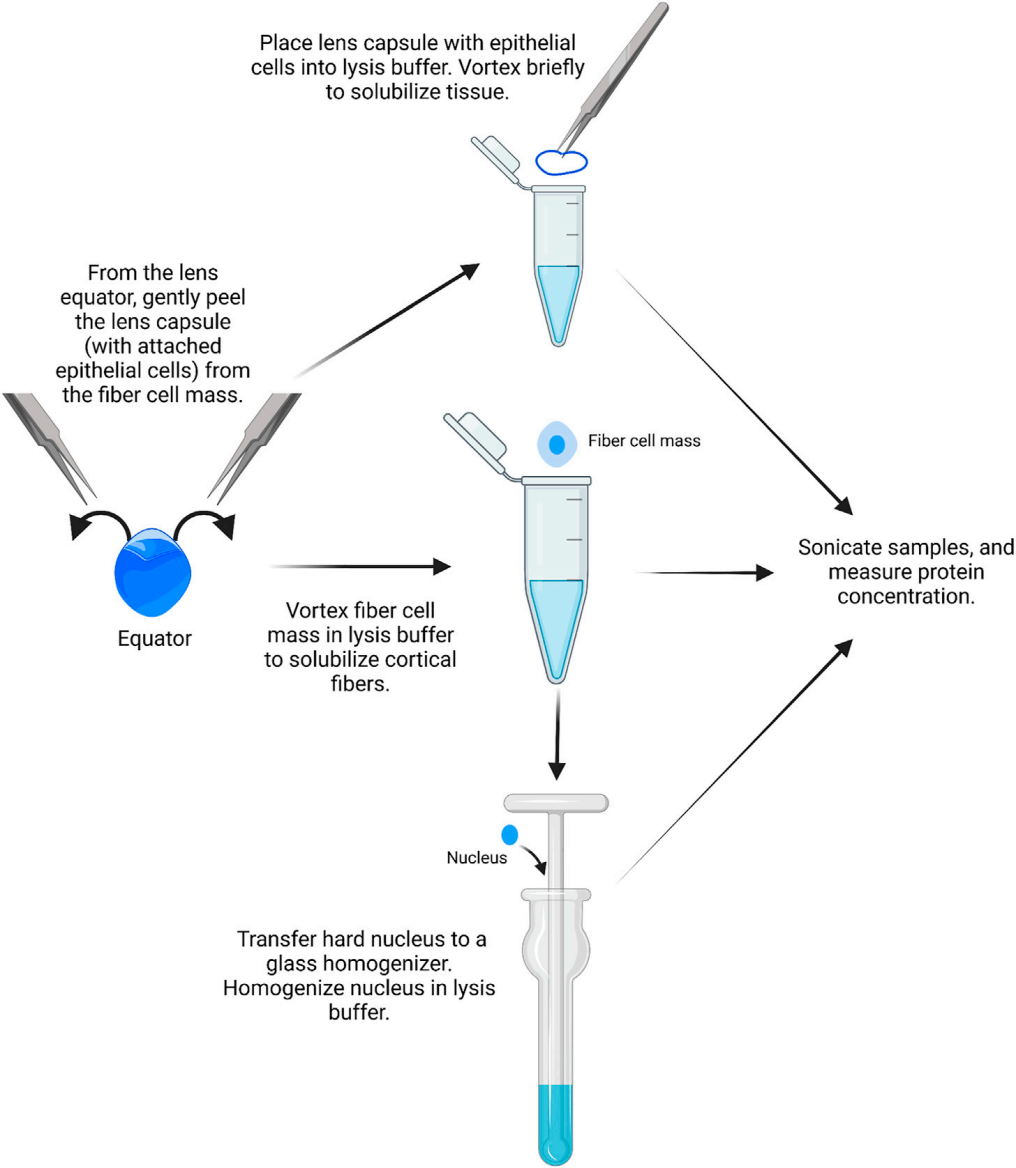
Epithelial, cortical fiber, and nuclear fiber protein sample isolation. Remove the lens capsule and isolate the protein sample from the lens epithelial cells. The fiber cell mass can then be vortexed to solubilize cortical fiber cells, leaving a hard lens nucleus. The hard lens nucleus can then be homogenized to create nuclear fiber protein lysates. The image was drawn using Biorender and not drawn to scale.

Similar to RNA isolation, it is possible to isolate proteins from single-lens capsules with attached epithelial cells, but the yield is very low. We recommend using pairs of lens capsules from one mouse to prepare the epithelial cell samples (Figure 3).

1) A pair of lens capsules/epithelium is placed into a 1.5-ml microcentrifuge tube filled with 16 μL of homogenization buffer (e.g., ice-cold lens homogenization buffer (Nowak et al., 2009; Gokhin et al., 2012; Cheng et al., 2018; Cheng et al., 2022) or 1X RIPA buffer) with phosphatase and protease inhibitors.

Lens homogenization buffer requires the addition of fresh 1 mM DTT. For epithelial cell samples, 14.75 µL lens homogenization buffer is mixed with 1.25 µL lens homogenization buffer with 2X sample buffer without bromophenol blue (see references above or Supplementary Table S1 for buffer contents). For fiber cell lysates, tissues should be homogenized in a 1:1 ratio of lens homogenization buffer and 2X sample buffer without bromophenol blue. 1X RIPA buffer and other commercial lysis buffers can also be used for homogenization. The ProteinSimple website should be referenced for buffer compatibility before starting. All homogenization buffers should be prepared fresh on the day of protein isolation.

2) The lens capsules are vortexed briefly in the homogenization buffer for two short pulses, and the tubes are centrifuged briefly in a mini table-top microcentrifuge.
3) Proteins from the lens cortex can be extracted from the lens fiber cell mass by placing a pair of dissected fiber masses into a 1.5-ml microcentrifuge tube filled with 250 μL of homogenization buffer per 10 mg of whole lens wet weight. The fiber cell masses are vortexed for 2–4 min in 30-second intervals until the ball of fiber cells does not become smaller in size with successive vortexing.

It should be noted that the leftover fiber cell mass is the compacted lens nucleus in mouse lenses.

4) Using clean, fine forceps, the pair of dense lens nuclei is transferred to a Dounce homogenizer (2 ml mortar, type B pestle) filled with 250 μL of homogenization buffer per 10 mg of the whole lens wet weight.
5) The lens nuclei are homogenized with the Dounce homogenizer until the solution is uniformly cloudy. Take caution to not get the lens tissue stuck at the bottom of the homogenizer to prevent loss of proteins.
6) The homogenized nuclear fiber cell lysates are transferred to microcentrifuge tubes using a gel-loading pipette tip. All samples are stored on ice before sonication.
7) The samples (QSonica Q55, 2 mm probe tip, amplitude 15) are sonicated in cycles of: 3 s of sonication and 10 s of cooling on ice. The epithelial samples are sonicated for one cycle, and the fiber cell samples are sonicated for three cycles.
8) The protein samples are stored at −80°C if not measuring the protein concentration immediately.
9) To determine the protein concentration, use the Bradford or bicinchoninic acid (BCA) assay as per the manufacturer’s instructions.

It should be noted that after measuring the protein concentration of epithelial cell lysates once, we can approximate the concentration of this fraction to conserve the amount of the protein sample. The amount of protein in epithelial cell samples from a pair of capsules from 6–8-week-old mice is ∼0.5 μg/μL. The amount of protein from cortical and nuclear fiber cells from a pair of lenses from 6–8-week-old mice is 6–10 μg μL and 4–6 μg/μL, respectively.

#### Protein sample preparation for JESS/WES capillary electrophoresis

The detection of proteins from pairs of lens epithelial cell samples requires the ProteinSimple JESS or WES capillary electrophoresis machine (Parreno et al., 2020; Cheng et al., 2022). It should be noted that all antibodies and protein concentrations should be titrated as per the manufacturer’s instructions prior to proceeding with actual experiments to determine the optimal conditions for antibody saturation and protein concentration. The following is a basic procedure for JESS/WES that does not cover the optimization protocol, assay setup, or data analyses. See discussion for more information on optimization, assay design, and data analysis.

1) The standard pack reagents are prepared as per the manufacturer’s instructions.
2) JESS/WES can identify the target protein at concentrations as low as 0.1 μg/μL. The epithelial cell samples are prepared by diluting 15 µL of the sample with 11.25 µL of 0.1X sample buffer provided by the manufacturer. For fiber cell lysates, samples are diluted to the appropriate concentration based on titration experiments with 0.1X sample buffer provided by the manufacturer in 1.5-ml microcentrifuge tubes. The samples will be mixed with one-part fluorescent 5X master mix to four parts of the prepared lysates.
3) Ensure that the samples are mixed by brief vortexing and spin down the tubes in a mini table-top microcentrifuge. The samples are incubated at 95°C for 5 min.
4) After incubation, the samples are briefly vortexed and spun down before placing them on ice until use.
5) Primary antibodies are diluted as appropriate in the diluent buffer provided by the manufacturer. The antibody diluent that is used will depend on whether the run requires chemiluminescence or fluorescence detection. If running fluorescence, a milk-free antibody diluent must be used to reduce the amount of background signal. Otherwise, antibody diluent 2 can be used.
6) The secondary antibody is prepared as per the manufacturer’s instructions. For chemiluminescence, the luminol-S and peroxide solutions are prepared as per the manufacturer's instructions, combining each in a 1:1 ratio.
7) For multiplexing on the JESS, the RePlex reagent is prepared as per the manufacturer’s instructions.
8) For these assays, we suggest using the total protein detection kit as the loading control according to the manufacturer’s instructions. This assay can be carried out using RePlex on the JESS or in separate wells on the WES. More details are provided in the discussion.
9) To load the capillary electrophoresis plate, the reagents are pipetted carefully according to Table 1. This is a generic example, so the plate assay design may vary depending on the experiment.
10) The plate is centrifuged at 1,000 × g for 5 min at room temperature before loading the wash buffer and RePlex buffer.
11) Set up the assay accordingly in the Compass for SW software. The plates take ∼3 h to run for standard assays and ∼5 h for RePlex assays.

**TABLE 1:** JESS/WES plate loading guide.

Well			
One antibody only	w/total protein	Component	Volume
A1	A1	Biotinylated ladder	5 μL
A2–A25	A2–A25	Lens lysate samples	3 μL
B1–B25, C1	B1, C1–C25, D1	Antibody diluent	10 μL
	B2–B25	Total protein labeling reagent	10 μL
C2–C25	D2–D25	Primary antibody	8.5 μL
D1	E1	Streptavidin-HRP	10 μL
D2–D25	E2–E25	Secondary antibody	10 μL
	F1–F25	Total protein streptavidin-HRP	8 μL
E2–E25	Row J	Luminol–peroxide	15 μL (immunoassay)
			170 μL (total protein)
Rows G, H, and I	Row G, H, I	Wash buffer	500 μL (load after step 10)
	Row K	RePlex reagent	300 μL (load after step 10)

#### Lens epithelial cell flat mounts and immunostaining

**FIGURE 4:**
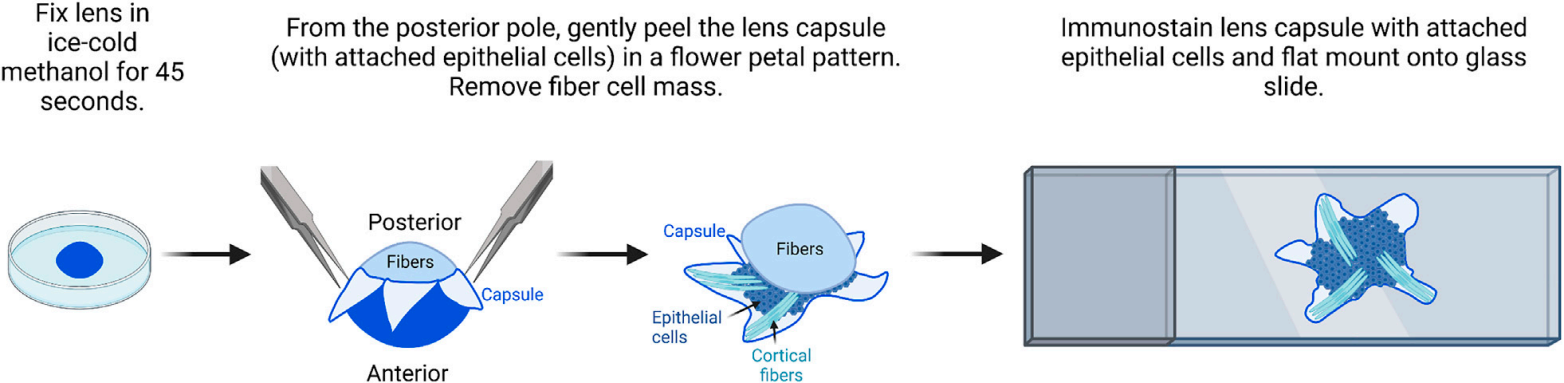
Flat mount imaging of lens epithelial cell monolayer. After methanol fixation of the lens, gently peel the lens capsule with attached epithelial cells from the posterior and remove the fiber cell mass. There will be a small amount of peripheral fiber cells attached to the flat mount sample. After immunostaining, flat mount the lens capsule onto a glass slide in mounting media and then cover and seal with a coverslip for imaging. The image was drawn using Biorender and not drawn to scale.

Mouse lens capsule flat mounts (Figure 4) (Cheng and Gong, 2011; Cheng et al., 2013; Cheng et al., 2017) were prepared using a protocol previously described for rat lenses (Sugiyama et al., 2010).

1) Lenses are dissected from freshly enucleated eyes and immediately fixed for 45 s in ice-cold methanol.

It should be noted for this procedure, the lens does not have to be perfectly clean of all extraneous tissues, but all larger tissue bits should be removed during dissection.

2) The fixed lens is transferred back into the dissection plate with 1X PBS. The anterior capsule will be whiter than the posterior capsule for a few seconds. The lens is oriented with the anterior lens capsule down toward the bottom of the dish.
3) Two pairs of fine forceps or one pair of fine forceps and one pair of dissection scissors are used to make a cross-pattern radial cut into the posterior lens capsule.
4) Fine forceps are used to peel the capsule petals toward the lens equator. The capsule flat mounts will contain epithelial cells and some superficial fiber cells.
5) Place the lens capsule flat mounts into a blocking solution (3–5% normal serum (species depends on the secondary) and 0.3% Triton X-100) for 1 h at room temperature. The blocking and immunostaining steps can be carried out in multi-well plates or 1.5-ml microcentrifuge tubes.
6) The lens capsule flat mounts are transferred to the primary antibody solution and incubated overnight at 4°C with gentle rocking.

The primary and secondary antibody concentrations should be optimized by immunostaining with lens frozen tissue sections.

7) The lens capsule flat mounts are washed with 1X PBS three times for 5 min per wash with gentle rocking.
8) The lens capsule flat mounts with appropriate fluorescent secondary antibodies are incubated for 2 h at room temperature with gentle rocking.

It should be noted that due to the methanol fixation, phalloidin dyes that stain F-actin networks will not work on these flat mounts.

9) The lens capsule flat mounts are washed with 1X PBS four times for 5 min per wash with gentle rocking.
10) On a clean SuperFrost Plus glass slide, place one generous dot (∼50 μL) of DAPI VectaShield mounting medium. Transfer one sample to the dot of the mounting media.
11) Two pairs of fine forceps are used to gently open up and flatten the lens capsules. If being imaged by confocal microscopy, since the tissue is not thick, any side can be facing up. If using a fluorescence microscope, the capsule should be side facing up. The sample does not have to be perfectly flat, but it should not be twisted or folded on itself.
12) A coverslip is placed on top of the flatten lens capsule. The weight of the coverslip will help the tissue become more flattened. For confocal microscopy, use 1.5-thickness coverslips.
13) Seal the coverslip with nail polish and allow it to dry overnight before imaging. Under the microscope, the epithelial cell side of the flat mount will usually have a few fiber cells stuck to it, while the capsule side will not.

## Method validation and discussion

### RNA isolation and primary culture epithelial cells

Extracting RNA from lens epithelium using TRIzol/chloroform-based precipitation requires pooling of multiple lens epithelial capsular peels for RNA processing. In our previous study, this required the pooling of at least six capsular peels (Parreno et al., 2020). Here we demonstrated, using a modified version of the TRIspin method previously developed for cartilage samples (Reno et al., 1997), that we are able to obtain enough RNA from just two lens capsules from 8−10-week old mice for real-time RT-PCR purposes. The RNA achieved A260/280 values of 1.875 with an average concentration of 131 ± 35.5 ng/μL (mean ± standard error; *n* = 3). To determine the quality of the extracted RNA, we measured the RNA integrity value (RIN) (Schroeder et al., 2006). We determined that our RIN from the lens epithelial cell samples was 5.0 ± 0.5, which is consistent with values from other tissues (Fleige and Pfaffl, 2006). This RIN value has been shown to be suitable for analyzing gene expression by real-time RT-PCR (Fleige and Pfaffl, 2006).

**FIGURE 5:**
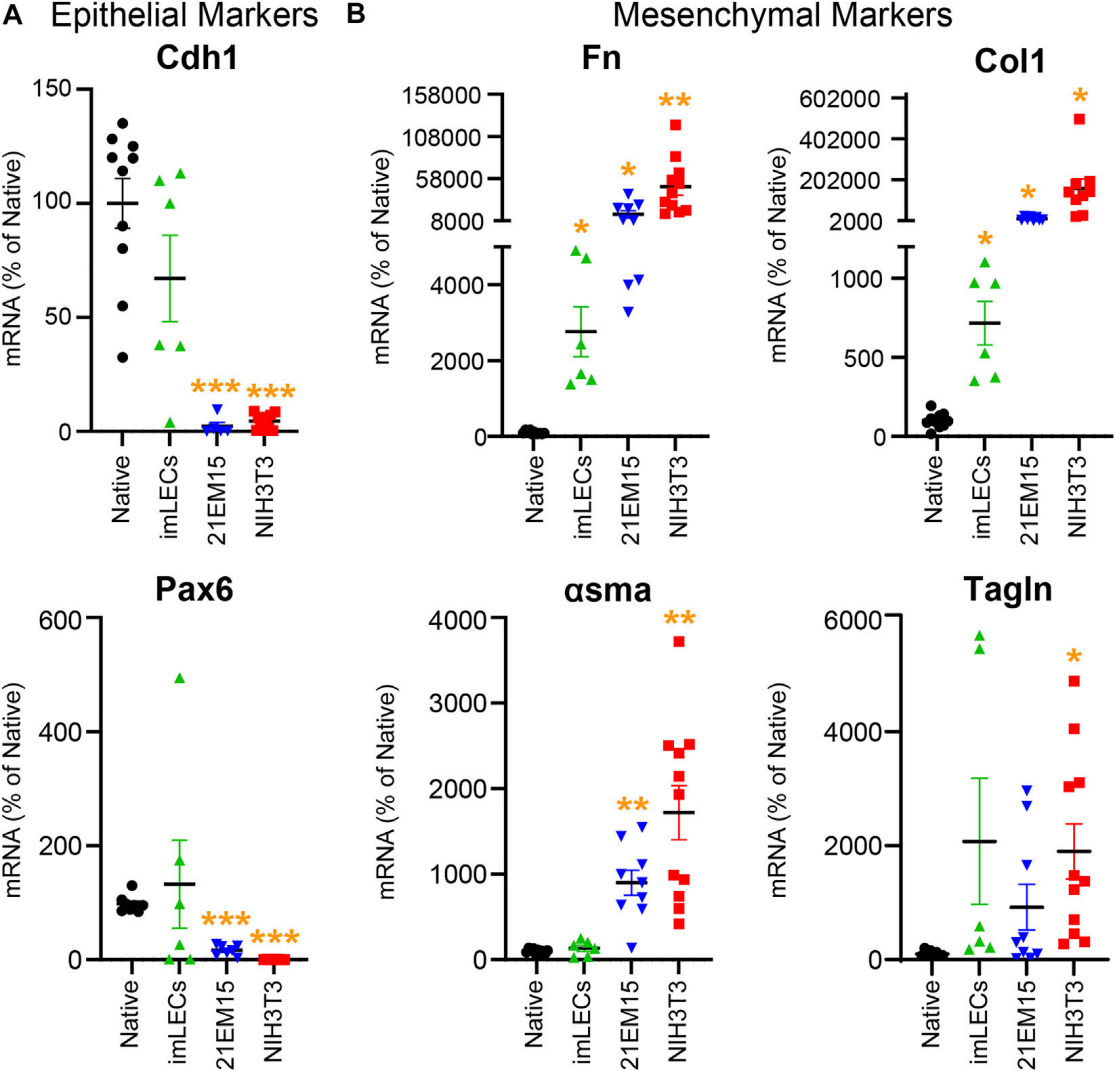
Analysis comparing the mRNA levels isolated from native mouse lens epithelial cells (Native), mouse immortalized lens epithelial cells (imLECs and 21EM15), and mouse fibroblasts (NIH3T3). **(A)** mRNA levels for the lens epithelial cell marker genes cadherin-1 (Cdh1) and paired box 6 (Pax6). While the imLEC cell line had no significant difference in the expression for epithelial cell markers compared to native cells, 21EM15 and NIH3T3 cells had significantly lower expression of epithelial cells markers than native lens epithelial cells. **(B)** mRNA levels for the mesenchymal/fibroblastic marker genes fibronectin (Fn), collagen-1 (Col1), α-smooth muscle actin (αsma), and transgelin (Tagln). The imLEC cell line had elevated expression for Fn and Col1 when compared to that of native lens epithelial cells. Both the 21EM15 and NIH3T3 cell lines had greater mesenchymal characteristics with substantially reduced epithelial marker mRNA levels and highly elevated mesenchymal/fibroblast marker mRNA levels. Dot plots show the average and standard error of mean. Individual genes were normalized to 18S. The mRNA levels were calculated using delta–delta Ct method and expressed as a percentage of native lens epithelial cell controls. *, *p* < 0.05; **, *p* < 0.01; ***, *p* < 0.001.

This RNA isolation technique allows comparison of gene expression between mouse native lens epithelial cells, immortalized lens epithelial cell lines (imLEC and 21EM15), and embryonic fibroblast cells (NIH3T3) (Figure 5). Our PCR analysis reveals that immortalized lens epithelial cell mRNA expression of epithelial cell markers does not fully resemble that of native lens epithelial cells. 21EM15 cells express significantly lower levels of epithelial markers, Cdh1 and Pax6, while having elevated mRNA levels for mesenchymal cell markers, Fn, Col1, and αsma, more closely resembling mRNA levels of NIH3T3 fibroblasts. While the epithelial marker expression in imLECs is similar to native lens epithelial cells, the expression of mesenchymal/fibroblast markers is elevated, including Fn and Col1. Based on these gene expression findings, caution must be exercised when using immortalized cell lines for experiments as they no longer fully resemble the native lens epithelial cells and express mesenchymal/fibroblastic markers.

Primary culture of lens epithelial cells has been performed by many different protocols on lenses from many species of animals. It is known that the isolated lens epithelial cells often lose their normal cuboidal shape, polarity, and cytoskeletal network when plated on hard tissue-culture substrates with media supplemented by FBS. Several groups have developed methods for serum-free primary culture (Wunderlich et al., 1994; Musil, 2012) to avoid changes and inconsistencies due to serum supplementation related to the batch of serum and the manufacturer. While it may be possible to preserve the morphology of primary culture lens epithelial cells through ECM modifications, such as laminin- or fibronectin-coated plates (Long et al., 2008; VanSlyke et al., 2018) or cultures on the native lens capsule (Campbell and McAvoy, 1984; McAvoy and Fernon, 1984; Nagineni and Bhat, 1988; 1989b; a;Saika et al., 2002; Wernecke et al., 2018), and through TGFβ inhibition (Wang et al., 2017), researchers should conduct a detailed study of RNA expression and cytoskeleton architecture, as we discuss below, before using cultured cells as models for native cells.

Though several issues make studying the primary culture or immortalized lens epithelial cells not an ideal representation for native lens epithelial cells, primary culture cells can be an excellent tool to understand the growth and behavior of transformed epithelial cells that occur during posterior capsular opacification (PCO) following cataract surgery. For PCO research, primary culture cells plated on various intraocular lens (IOL) materials could reveal methods to prevent unwanted cell adhesion onto the surface of IOLs that are inserted during cataract surgery to replace the native lens. Several groups have also cultured primary lens epithelial cells on the lens capsule to maintain a normal ECM environment and mimic the proliferation and migration of the epithelial cells after cataract surgery onto the posterior lens capsule (Liu et al., 1996; Wormstone et al., 1997; Saxby et al., 1998; Futter et al., 2005; Zelenka et al., 2009; Andjelic et al., 2014; Sundelin et al., 2014; Luft et al., 2015; Recek et al., 2016). Thus, while the use of primary culture or immortalized epithelial cells is not an ideal model for native epithelial cells, the isolation of these cells can be used to advance our understanding of pathological changes that occur after cataract surgery and may be an appropriate model for testing new PCO treatments.

### Protein separation *via* capillary electrophoresis

**FIGURE 6:**
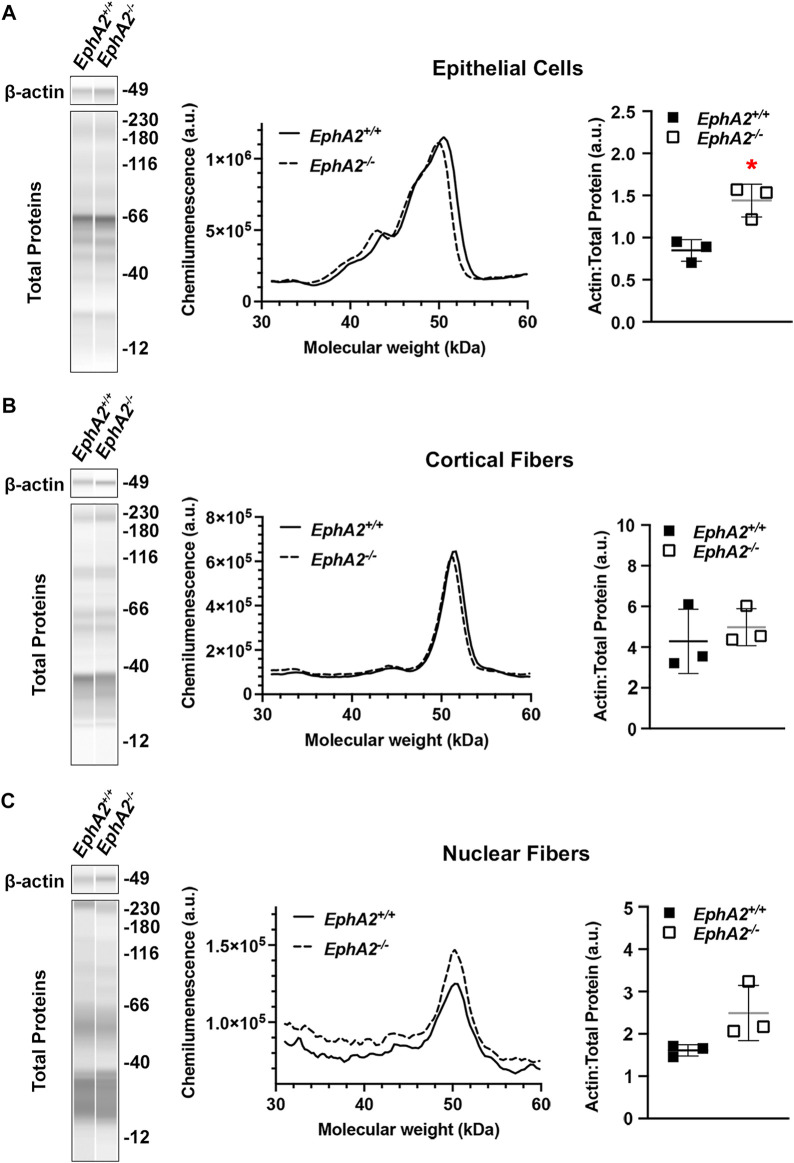
Analysis of epithelial, cortical fiber, and nuclear fiber protein samples by capillary electrophoresis. Representative gel bands for β-actin (∼49 kDa) and total protein profiles (12–230 kDa) from each fraction are presented in pseudo-lane views. Normal wild-type (+/+) control lenses are compared to knockout (−/−) lenses. All mice were littermates and 4 months old. **(A)** Representative electropherogram of β-actin peaks from the epithelial cell fraction is plotted for control (+/+) and knockout (−/−) samples. The β-actin protein amount normalized to total protein is plotted on the dot plots with lines showing the average and standard deviation. The knockout samples showed increased amount of β-actin in the epithelial cells. *, *p* < 0.05. **(B,C)** Representative electropherograms of β-actin in cortical fibers and nuclear fibers from control and knockout lenses. Dot plots show the average and standard deviation for the normalized amount of β-actin in each sample. There is no difference in β-actin levels in control vs. knockout lenses in the cortical fiber and nuclear fiber fractions.

Traditional methods for Western blotting using SDS-PAGE gels and membrane transfer require high protein concentrations (>15–20 μg/μL) and volume of sample (15–20 µL). The monolayers of lens epithelial cells yield relatively low protein concentration, and thus, excessive numbers of lens epithelial cell samples would need to be pooled together to run traditional Western blots. The capillary electrophoresis systems (JESS/WES) by ProteinSimple have made it possible to utilize a pair of lens epithelial cell samples to test several (3–5) antibodies (Parreno et al., 2020; Cheng et al., 2022). We recently published our results for detecting EphA2 in whole lenses, lens epithelial cells, cortical fiber cells, and the lens nucleus using this method (Cheng et al., 2022), and we demonstrate in Figure 6 that the same method can be applied to determine the levels of β-actin different compartments of the lens. Protein lysates from other monolayers of cells in the eye, including the corneal endothelium (Ogando et al., 2021; Shyam et al., 2021), retinal pigmented epithelial cells (Vessey et al., 2022), and from embryonic lens samples (Gheyas et al., 2022), can also be used on this platform.

In this protocol, we have also included the method to prepare the cortical fiber cell and nuclear fiber cell protein lysates (Figures 3, 6). For nuclear fiber lysates, pilot studies may be needed to determine the normal size of the lens nucleus to estimate the duration of vortexing to remove all cortical fiber cells. To serve as a guide, we have previously measured the lens nucleus size in C57BL/6 J wild-type lenses from mice of various ages (Cheng et al., 2019). The nucleus of rodent lenses is firm and can be easily removed by mechanical disruption (Cheng et al., 2016). For mutant lenses that may have compromised fiber cells, lens nucleus separation may be more challenging and require careful handling (Cheng et al., 2022). In those cases where the lens nucleus cannot be easily removed, the entire lens cell mass may be homogenized together in the Dounce homogenizer for a total fiber cell fraction.

Assay design and optimization of protein and antibody concentrations are required to produce the best results. ProteinSimple offers free video tutorials on assay design, plate loading, and detailed data analysis through the BioTechne Academy (https://academy.bio-techne.com/learn/signin). In general, we would recommend titration of every new antibody with appropriate negative control when possible and utilization of the total protein detection kits as the loading control. Using the total protein detection as the loading control avoids arbitrary designation of a housekeeping protein that may be inadvertently and/or unknowingly be altered between the test and control samples (Aldridge et al., 2008; Moritz, 2017; Wang et al., 2021). In the JESS system, RePlex allows for total protein to be detected after stripping and re-probing steps within the same capillary used for target detection. This allows for protein signal normalization within each capillary to minimize loading and sample preparation variability.

### Visualizing of lens epithelial cell structures in cell culture, flat, and whole mounts

**FIGURE 7:**
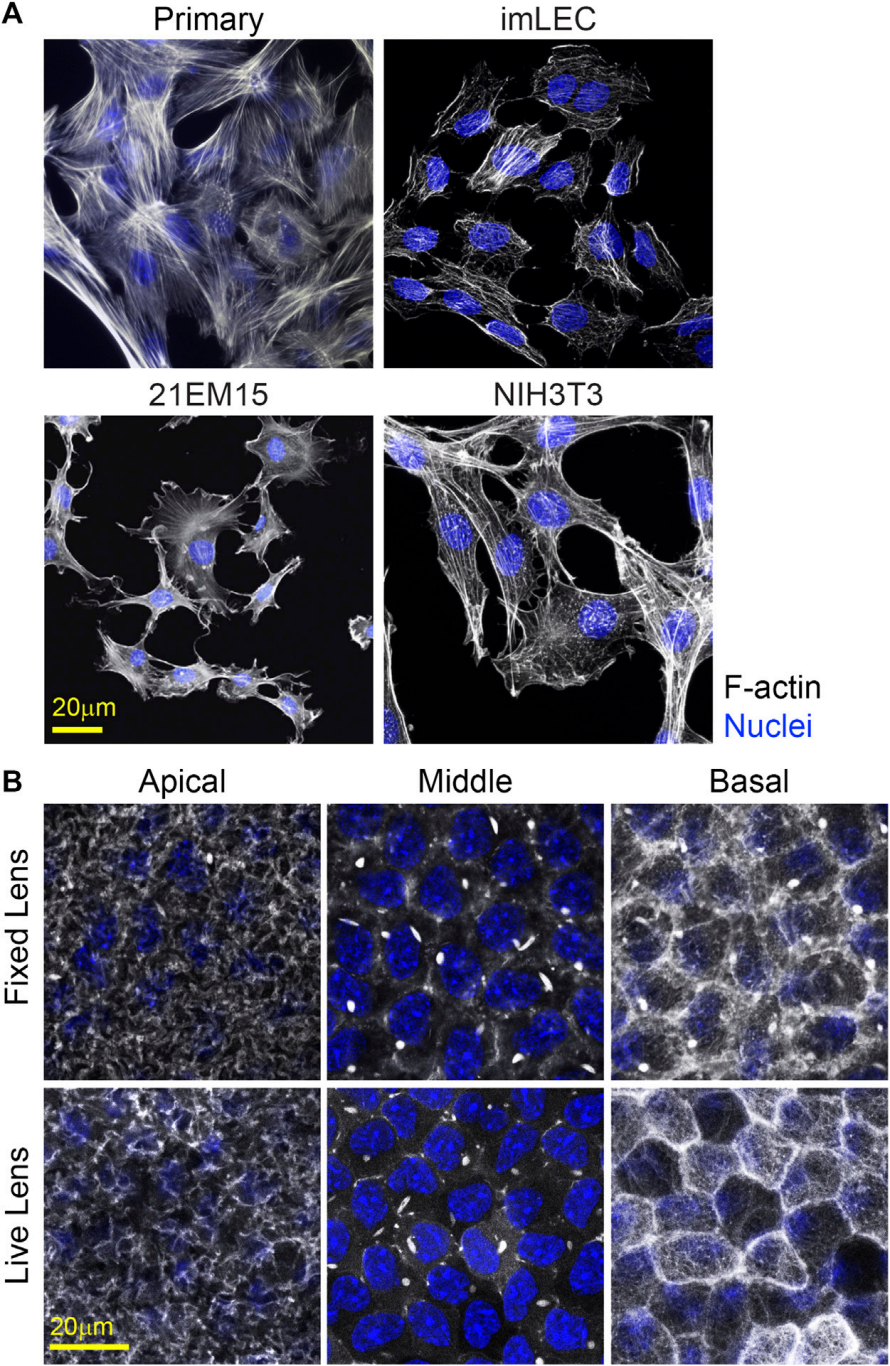
F-actin staining for cultured cells and in whole mount imaging of F-actin in fixed and unfixed lenses. **(A)** Cells were stained for F-actin (light gray) and nuclei (blue). Primary lens epithelial cells cultured after 7 days of initial seeding on glass dishes contain stress fibers similar to imLECS, 21EM15, and NIH3T3 cells in culture. **(B)** C57BL/6J wild-type fixed mouse lenses or LifeAct-GFP transgenic unfixed mouse lenses imaged for F-actin (light gray) and nuclei (blue). Fixed lenses (wild-type) were stained for F-actin using phalloidin, while unfixed lenses expressed LifeAct-GFP, allowing for visualization of F-actin directly. The epithelial cells nuclei in both fixed and unfixed lenses were stained with Hoechst. Images shown are of single optical slices. Both fixed and unfixed lens anterior epithelial cells had similar basal reticular F-actin organization with limited stress fibers. At the middle region in the lateral sides of these cuboidal cells, F-actin is at cell–cell junctions and within the bright structures called sequestered actin bundles (SABs). The basal region of fixed lens epithelial cells differed from that in unfixed lenses. In the unfixed lens, the basal region of lens epithelial cells had polygonal actin arrays, while, in the fixed lens, the polygonal arrays appear disrupted on the basal surface of these epithelial cells. Furthermore, SABs were present in the basal region of fixed lens epithelial cells. Scale bars, 20 μm.

**FIGURE 8:**
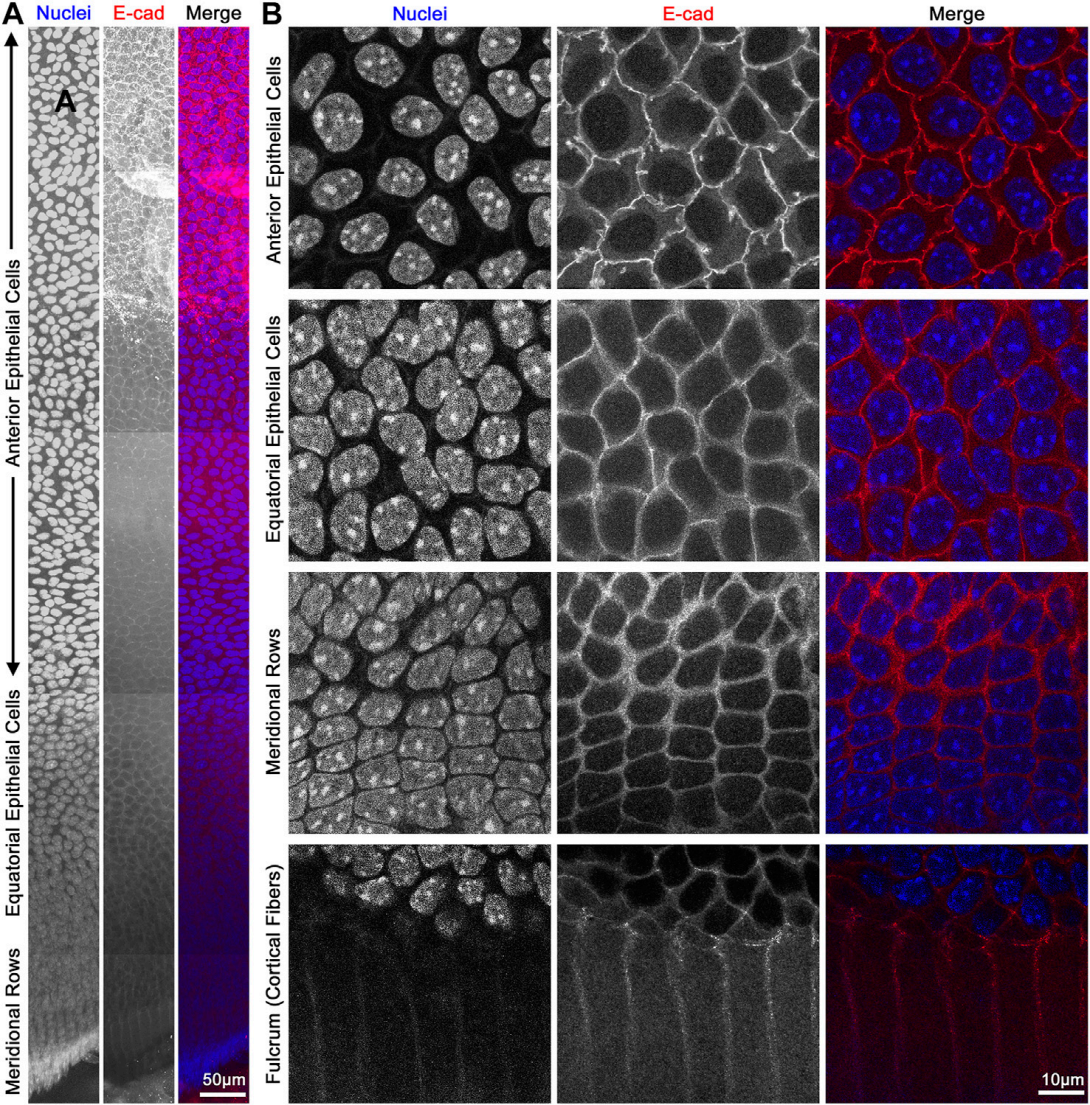
Flat mount of lens epithelial cell monolayer. **(A)** Lens capsule flat mount was stained for E-cadherin (red) and nuclei (blue). Tiled Z-stack scans from the anterior (top) to the meridional rows at the equator (bottom) were stitched, and this image is a maximum intensity projection of the stitched Z-stacks compressed into a 2D image. Scale bar, 50 μm. **(B)** Magnified images of epithelial cells from different regions of the lens. As expected, E-cadherin outlines the membrane of the epithelial cells and has reduced signals after epithelial cells differentiate into fiber cells at the lens fulcrum. It should be noted that the spacing of the nuclei changes slightly between anterior vs. equatorial epithelial cells. Scale bar, 10 μm.

Visualization of cell proteins/structure in primary and immortalized lens epithelial cells is possible using traditional fluorescence immunostaining techniques (Figure 7A). However, primary and immortalized lens epithelial cell shape and cytoskeletal structure are markedly different from native lens epithelial as visualized by flat (Figure 8) or whole mounting (Figure 7B). Unlike native lens epithelial cells, cultured primary and immortalized epithelial cells are no longer attached to one another. Furthermore, cultured epithelial cells contain F-actin organized into stress fibers unlike native lens epithelial cells, further supporting the notion that cultured lens epithelial cells have fibroblastic characteristics (Parreno et al., 2020).

The monolayer of lens epithelial cells is difficult to visualize in the lens tissue sections as we can only visualize a cross section of those cells. Lens capsule flat mounts with attached epithelial cells and a thin layer of peripheral lens fibers allow for examination of the entire monolayer of epithelial cells from different areas of the lens, anterior cells, proliferating equatorial cells, and differentiating equatorial cells that are packed into meridional rows (Figure 8). This method permits the study of the basal surface adjacent to the lens capsule, the lateral membrane between the epithelial cells, and the apical surface juxtaposed by the apical tips of newly formed lens fibers. The anterior epithelial cells are cobblestone in cross section, evenly spaced, and similar in size with oval nuclei. In contrast, cells closer to the lens equators are more closely packed and retain the cobblestone shape. As the equatorial epithelial cells start to differentiate, the cells become hexagon-shaped and organized into meridional rows. These cells are hexagonal on the basal lateral sides and narrow to the point at the apical surface and anchor at the fulcrum (Sugiyama et al., 2009) or modiolus (Zampighi et al., 2000) before continuing their elongation and differentiation programming to become lens fiber cells.

Flat mount preparations do not always have continuous and wrinkle-free regions, and thus, using the epithelial cell shape and arrangement as a guide is an important visual landmark to determine the approximate region of the epithelial monolayer being observed. A consideration when collecting Z-stacks through epithelial cells is axial distortion (Smith et al., 2018). Oversampling in the *Z*-axis is needed to correct the stretch distortion for proper 3D reconstructions (Smith et al., 2018). Flat mount preparations from rat, pig, or human lenses have also been prepared by pinning the capsule with epithelial cells into a flat plate (Zelenka et al., 2009; Wu et al., 2015; Kumar et al., 2019). But in our experience, it is difficult to pin the capsule to a plate with small mouse lenses, and fixation of mouse lenses with paraformaldehyde and then peeling the lens capsule result in thin and completely transparent samples that are fragile and easily damaged during the immunostaining process. There are examples of capsular preparation from mouse lenses where small pieces of the lens capsule are adhered to poly-lysine-coated slides (Liou and Rafferty, 1988; Rafferty and Scholz, 1989). These preparations, however, would only allow the study of small regions of the epithelial monolayer.

Drawbacks of flat mounts include the loss of 3D structure of the lens and the disruption of apical–apical epithelial-to-fiber cell connections due to mechanical dissection. An alternate method to visualize lens epithelial cell structures is to perform whole lens staining. Small dyes, including wheat germ agglutinin (WGA) or phalloidin, can penetrate the lens to stain cell membranes or F-actin network, respectively (Cheng et al., 2013; Parreno et al., 2018; Cheng et al., 2019). However, large antibodies do not readily penetrate the lens capsule. We have previously detailed a method to immunostain the whole lens after incubation in collagenase to partially digest the lens capsule and facilitate antibody penetration (Patel et al., 2021). Whole mount staining maintains the 3D structure of tissue, but imaging whole mount lenses requires repositioning of the lens to visualize the various regions of the epithelium. Excessive disruption of the lens capsule for antibody labeling may alter the basal surface of lens epithelial cells and lead to cellular disorganization (Patel et al., 2021).

An alternative to imaging of fixed and stained lenses is performing confocal imaging on unfixed lenses. Previously, we examined the effect of whole lens compression on epithelial morphology using lenses from mice that express tdTomato, which localizes to cellular membranes (Parreno et al., 2018). In this study, we extend our unfixed lens imaging to visualization of cytoskeletal F-actin structures using lenses from LifeAct-GFP transgenic mice. F-actin networks in unfixed epithelial cells in transgenic LifeAct-GFP lenses resemble those of fixed whole lenses stained with phalloidin (Figure 7B). However, the fixation may affect cell structures and disrupt the F-actin network. For instance, in fixed whole lenses, the sequestered actin bundles (SABs) (Rafferty and Scholz, 1984; Rafferty, 1985; Rafferty and Scholz, 1985; Scholz and Rafferty, 1988; Rafferty and Scholz, 1989; Rafferty et al., 1990) are bright structures that appear at the basolateral regions of the lens epithelial cells. In contrast, SABs are only found in the lateral (middle) regions of the unfixed lenses. Thus, the fixation conditions for whole lens imaging may need to be optimized to best preserve the native cell morphology and cytoskeletal arrangements.

## Conclusion

Even though the monolayer of epithelial cells in the lens forms just a small fraction of the entire lens tissue, it plays a principal role in lifelong lens growth and pathological conditions (i.e., PCO formation after cataract surgery). The protocols described here can facilitate the enrichment of epithelial RNA and protein, allowing for the study of molecular expression without pooling of large numbers of lenses. The protocols will need further refinement to increase the RIN for RNA-seq experiments, and future developments in technology may allow proteomic analysis on low volume epithelial cell protein lysates. Furthermore, the imaging techniques will permit investigations of the lens epithelial cellular structure in flat mounts, fixed lenses, or unfixed lenses, which more faithfully retain the native lens epithelial cell structure. The development of new transgenic mouse lines with fluorescent markers may allow more detailed imaging of unfixed whole lenses. Future studies may also compare ultrastructure information from electron microscopy images with whole lens or flat mount staining. Our detailed protocols allow for the study of native epithelial cell biology and the mechanistic role(s) they play in pathologies.

## Data Availability

The raw data supporting the conclusion of this article will be made available by the authors, without undue reservation.
